# Effect of sodium hypochlorite temperature and concentration on the fracture resistance of root dentin

**DOI:** 10.1186/s12903-024-03954-y

**Published:** 2024-02-13

**Authors:** Reem M. Barakat, Rahaf A. Almohareb, Munirah Alsuwaidan, Ebtihal Faqehi, Enas Alaidarous, Fahda N. Algahtani

**Affiliations:** 1 Dental Clinics Department, King Abdullah bin Abdulaziz University Hospital, Princess Nourah Bint Abdulrahman University, Box 84428, Riyadh, 11671 Saudi Arabia; 2 Department of Clinical Dental Sciences, College of Dentistry, Princess Nourah Bint Abdulrahman University, P.O. Box 84428, Riyadh, 11671 Saudi Arabia; 3 Dental Intern, College of Dentistry, Princess Nourah Bint Abdulrahman University, P.O. Box 84428, Riyadh, 1167 Saudi Arabia

**Keywords:** Dentin, Fracture resistance, Irrigation, Root, Sodium Hypochlorite

## Abstract

**Background:**

Sodium hypochlorite (NaOCl) is the most efficient root canal irrigant to date. The aim of this study was to compare the effect of NaOCl used at different temperatures and concentrations on the compressive strength of root dentin.

**Materials and methods:**

Seventy-two extracted human single-canaled straight roots of comparable size and length were selected and randomly divided into six groups (*n* = 12): Group (A) served as a control with unprepared canals. The other groups were instrumented with rotary ProTaper Universal files up to size F3. Group (B) was irrigated with 1% NaOCl at room temperature, Group (C) with 1% NaOCl heated to 70 °C, Group (D) with 5.25% NaOCl at room temperature, and Group (E) with 5.25% NaOCl heated to 70 °C. Saline was used in Group (F). The roots were sectioned into 2-mm-thick disks that underwent compression testing using a universal testing machine. Data were analyzed using one-way ANOVA and *post hoc* Tukey tests. The significance level was set at *p* ≤ 0.05.

**Results:**

A total of 255 disks were tested. The control group showed the highest compressive strength (*p* = 0.0112). However, this did not differ significantly from that of heated (*p* = 0.259) or unheated (*p* = 0.548) 1% NaOCl. There were no statistically significant differences between the groups of instrumented teeth.

**Conclusion:**

Within the conditions of this study, irrigation with NaOCl at different concentrations and temperatures during root canal preparation did not affect the compressive strength of root dentin. Clinical Relevance: This study demonstrates that the use of NaOCl as a root canal irrigant is not associated with a clinically relevant decrease in root compressive strength, especially when compared to saline.

**Supplementary Information:**

The online version contains supplementary material available at 10.1186/s12903-024-03954-y.

## Background

Due to the anatomical complexity of the root canal system, successful endodontic treatment requires a combination of mechanical and chemical preparation to remove infected tissue and achieve effective disinfection of the root canals. To date, sodium hypochlorite (NaOCl) remains the preferred chemical irrigant due to its broad-spectrum antibacterial properties against species involved in endodontic infection, such as *Enterococcus faecalis*, *Actinomyces naeslundii*, and *Candida albicans* [1], in addition to its ability to dissolve necrotic tissue.

NaOCl can dissolve amino and fatty acids, leading to the formation of chloramine, which disrupts bacterial cell metabolism by inhibiting bacterial enzymes. Additionally, it leads to an elevated pH exceeding 11. This alters cytoplasmic membrane integrity, causing bacterial cell death [2].

These effects are not limited to bacterial cells. Almost a quarter of dentin weight consists of organic material, which is mostly type I collagen [3]. Studies have reported that NaOCl causes degeneration of dentin’s collagen structure and alters the expression of matrix metalloproteinases within it [4]. This, in turn, affects dentinal microhardness and bond strength [5].

Endodontically treated teeth are susceptible to fracture. Although this weakening appears to stem from the loss of tooth structure as a result of caries, access cavity preparation, and instrumentation of the root canal, much research has investigated the effect of NaOCl irrigation on dentin strength. The results remain inconclusive [6], with some studies highlighting the effect of NaOCl concentration on dentin resistance to fracture [6–8].

NaOCl concentrations ranging from 0.5 to 6% are used in endodontic treatment. Using greater concentrations allows higher effectiveness at eradicating bacterial biofilm from the root canal. However, it also entails an elevated risk of cytotoxicity, should the solution extrude into the periapical tissue. To ensure efficacy at lower concentrations, many methods have been suggested, such as applying ultrasonic vibration to the solution, prolonging working time, and heating the irrigation solution. Pre-heated NaOCl allows faster tissue dissolution coupled with enhanced antibacterial properties without affecting its short-term stability [9, 10]. While some have debated the feasibility of sustaining heated NaOCl delivery within the root canal system [11], extra- and intra-radicular heating techniques have been shown to be effective [12, 13]. It is possible to increase and maintain the temperature of intracanal NaOCl solution at a temperature of 40–49 °C by placing a heat-generating plugger and applying heat cycles of 5–10 s [11]. Heating NaOCl extra-orally to 70 °C was found to result in an intracanal irrigant temperature increase to 44 ± 2.5 °C [12].

Previous studies have explored the effect of heated NaOCl on the mechanical properties of dentin. In these studies, dentin bars were prepared and totally immersed within the solution [14, 15]. This methodology does not replicate the clinical scenario, during which NaOCl irrigation contacts only the internal dentinal surface of the root canal. A recent study employed a simple study design that mimicked the clinical situation and explored the effect of different concentrations of NaOCl on dentin microhardness. It concluded that 1% NaOCl was the safest concentration [7]. Since heating renders NaOCl more effective [9, 10], it would be interesting to further explore the effect of heating NaOCl on fracture resistance and whether heated NaOCl of a certain concentration is as safe as unheated NaOCl. Therefore, this study aimed to assess the effect of root canal irrigation with heated NaOCl at different concentrations on the fracture resistance of the dentin of prepared root canals. The null hypothesis was that there is no difference in the load required to fracture dentin exposed to different concentrations of heated and unheated NaOCl.

## Materials and methods

This ex vivo study received an exemption from the Internal Review Board of Princess Nourah bint Abdulrahman University (IRB no. 22–0444). Seventy-two human permanent teeth with single, straight canals were extracted after obtaining informed consent. Teeth with root caries, previous endodontic treatment, or excessively long or short roots were excluded. Samples were stored in a phosphate-buffered saline solution and placed in an incubator at 37 °C until use. G*Power 3.1 software (Heinrich-Heine-Universität, Düsseldorf, Germany) was used to calculate the sample size. The power was estimated as 90%, the probability of Type 1 error or α as 0.05, with an effect size (*f) =* 0.05.

### Sample preparation

**Fig. 1:**
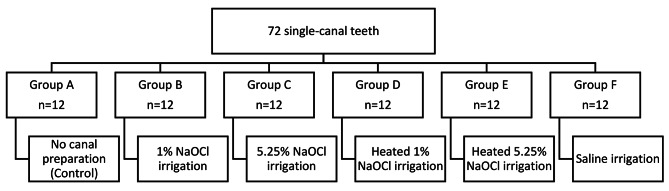
Distribution of sample teeth among the different experimental study groups

The crowns of the teeth were sectioned using a high-speed handpiece. The roots were arranged according to size and length, forming six groups containing similar combinations of root widths with standardized lengths (16 mm). Group (A) was established as a control group (*n* = 12) and did not undergo any root canal preparation. The remaining roots were instrumented using ProTaper Universal nickel titanium rotary files (Dentsply Sirona, Charlotte, NC, USA) mounted on a 6:1 reduction contra-angle handpiece connected to a rotary electric endo motor (X-Smart, Dentsply Sirona, Charlotte, NC, USA). Files were used according to manufacturer instructions up to size F3. Depending on the group, the canals were irrigated with either saline or NaOCl at different concentrations and temperatures (Fig. 1). Group (B) roots were irrigated during preparation with room temperature 1% NaOCl, and Group (C) roots were irrigated with 5.25% NaOCl at room temperature. Group (D) and (E) roots were irrigated with heated 1% NaOCl and 5.25% NaOCl, respectively. Finally, Group (F) roots were irrigated with phosphate-buffered saline. A water bath equipped with a thermometer was used to heat NaOCl to 70 °C. This temperature was selected based on the results of a previous study that highlighted this temperature as capable of sustaining an increase in the temperature of the intracanal solution [12].

Irrigation was performed with a 27-gauge needle using 1 mL of the irrigant before and after every rotary file. The irrigant was left within the canal during instrumentation. All canals received a final irrigation of 1 mL of ethylenediaminetetraacetic acid (EDTA) alternated with 1 mL of saline.

### Compressive force testing

**Fig. 2:**
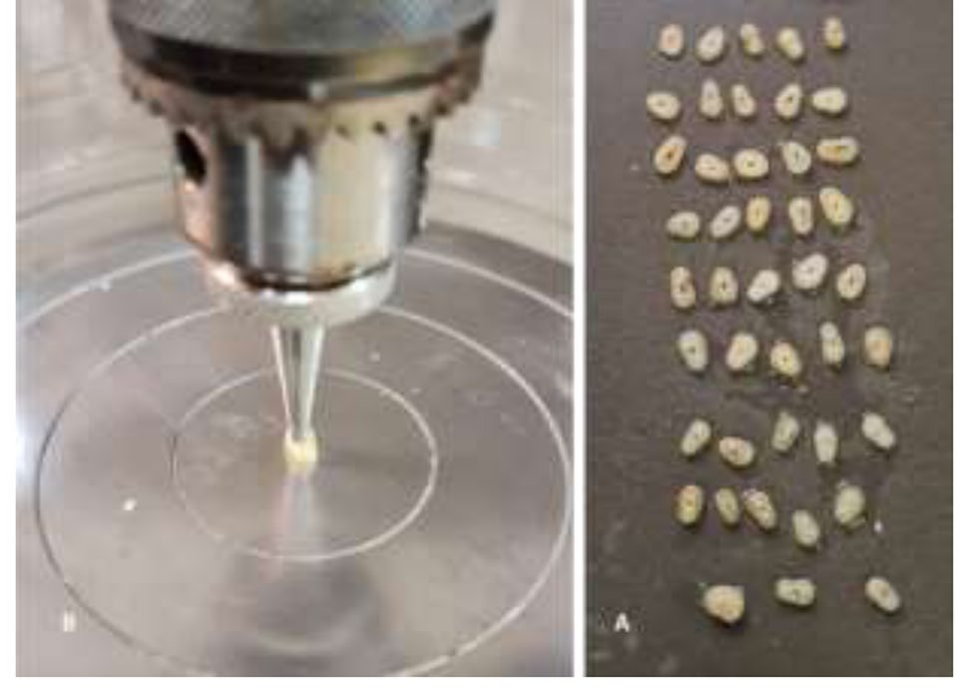
**(A)** Dentinal disks from one of the experimental groups undergoing compressive strength testing **(B)**

The roots were cut into 2-mm-thick disks using a Buehler Isomet low-speed saw with water cooling by an operator who was blinded to the groupings. Disks with observable cracks led to the exclusion of all disks from the respective tooth. Samples were then stored in an incubator (Raypa, Incuterm Digit, Terrassa, Spain) at 37 °C for 24 h. Each specimen was mounted on a support stage, and a downward compressive force was applied using a universal testing machine (Instron testing machine, Model 5967, ITW, MA, USA). A 2-mm-diameter rod was centered on the root canal and applied at a rate of 0.5 mm/min. The maximum load to fracture was registered in Newton (Fig. 2).

### Statistical data analysis

Data were analyzed using SPSS software version 22 (SPSS Inc., Chicago, IL, USA). Data normality was checked using the Shapiro–Wilk test, then one-way analysis of variance (ANOVA), followed by Tukey’s *post hoc* tests, was performed. A significance level of *p* ≤ 0.05 was selected.

## Results

A total of 255 disks were subjected to compressive testing. Table 1 shows the means and standard deviations for the forces required for failure in all groups. The non-instrumented (control) group showed the highest compressive strength. This was statistically significantly higher when compared to all the other instrumented groups (*p* = 0.0112) except for those irrigated with heated 1% NaOCl (*p* = 0.259) and 1% NaOCl at room temperature (*p* = 0.548).

There was no statistically significant difference between the strength of instrumented teeth irrigated with different solutions at different concentrations and temperatures (Table 1).

**Table 1:** Mean force of fracture of the different groups

Group		N	Mean force (N)	Standard Deviation
A	Control	40	638.99^a^	± 273.69
B	1% NaOCl	43	550.05^a,b^	± 208.30
C	5.25% NaOCl	43	468.49^b^	± 290.93
D	Heated 1% NaOCl	43	524.37^a,b^	± 216.92
E	Heated 5.25% NaOCl	43	475.16^b^	± 262.39
F	Saline	43	477.64^b^	± 178.96

Different letters indicate significant differences, with a *p*-value ≤ 0.05

## Discussion

NaOCl possesses characteristics of an ideal root canal irrigant, such as the ability to dissolve organic tissue, strong antimicrobial efficacy, long shelf life, and affordability [4]. However, it also has many disadvantages, most notably, elevated cytotoxicity, inability to remove the smear layer, and its effect on the physical properties of root dentin. The smear layer created during root canal instrumentation acts as a barrier, blocking the dentinal tubules, restricting the penetration of NaOCl, and impeding its disinfection capabilities [16]. While NaOCl can eliminate the organic part of the smear layer, it proves ineffective against the inorganic component. EDTA, on the other hand, removes this inorganic part. In this study, EDTA was employed as a final rinse, which is a recommended protocol for EDTA removal [17].

In root canal treatment, NaOCl is used in concentrations ranging from 0.5 to 6%. However, there has been much debate over the ideal concentration of NaOCl that balances its advantages and disadvantages. One issue that has received special interest is the effect of NaOCl on dentin composition, its mechanical and physical properties, and subsequent tooth resistance to fracture. Two studies reported that exposure to 5% NaOCl for an extended period of time was associated with the appearance of cracks on the surface of dentin [18, 19]. Dentin permeability was also altered by exposure to an increased concentration of NaOCl, according to Marending et al. [18].

In the present study, the effect of NaOCl concentration and temperature on dentin compressive strength was investigated. The non-instrumented (control) group showed the highest compressive strength. This was in accordance with a similar study that showed that root canal preparation had the most significant effect on root dentinal strength, irrespective of the irrigant [7]. In that study, NaOCl was used in various concentrations ranging from 1 to 10%. However, only 1% NaOCl was associated with greater dentinal strength than the higher concentrations, and there were no significant differences among the higher concentrations. For this reason, the present study aimed to compare NaOCl at two concentrations: 1% and 5.25%.

An interesting finding was that the decrease in compressive strength following 5.25% irrigation was not statistically significantly different from that observed with 1% NaOCl. Additionally, the decrease in strength after irrigation with 1% NaOCl was not statistically significantly different from that observed in the unprepared root canals. In contrast, the saline group showed lower compressive strength than the 1% NaOCl group, although again this was not statistically significant. This is in accordance with a study by Li et al. [7] and another study that reported a decrease, although not statistically significant, of the flexure modulus of dentin irrigated with saline compared to 2% NaOCl [20]. A possible explanation is that the introduction of saline into the root canal system during a single irrigation cycle might achieve the maximum specific tooth surface strain limit, which would lead to an adverse effect on the subsequent loading strain [21].

Previous studies testing the effect of NaOCl on dentin strength have been conducted by immersing specimens in NaOCl solution rather than using NaOCl as a root canal irrigant [14, 15]. The results of such studies are clinically irrelevant, as the entire specimen is subjected to NaOCl, rather than just the internal dentinal root canal wall. Recently, a new and simple method of specimen preparation was suggested, whereby roots prepared and exposed to the irrigant were sectioned into thin disks [6]. Compressive forces were then applied to the disks via a metal rod inserted into the prepared canal. This technique allowed the simulation of the forces to which a root would be exposed during clinical treatment, for example, post insertion [6]. The present study employed this methodology and replicated the clinical scenario in that it exposed only the canal dentinal walls to NaOCl and limited the exposure time to that of an average clinical session. However, the rod used in the present study was larger in diameter and exerted forces beyond the inner canal walls.

In a study examining the effect of irrigation solution on dentin microhardness, in which only the internal dentin surfaces of the root canals were subjected to irrigants in a similar manner to this study, the decrease in dentin microhardness with increased NaOCl concentration was limited to a depth of only 0.35–0.4 mm, but an increase in NaOCl concentration did not lead to an increase in the extent of this depth [6].

Dentin is composed of 22% organic material, which is mainly type I collagen fibrils, encapsulated within inorganic apatite crystals. This collagen structure promotes dentin viscoelasticity, toughness, and fatigue resistance [22]. The dissolution of collagen fibers is affected not only by the NaOCl concentration but also by its temperature. A previous study measured the rate of collagen fiber dissolution and found it comparable between 5% NaOCl solution at 20 °C and 1% NaOCl solution at 36 °C [23]. When heated to 60 and 80 °C, NaOCl has been reported to affect the collagen and mineral dentin interface, reducing its elasticity and delaying recovery following loading, thereby promoting microcracking.

Previous studies reported that heating NaOCl above room temperature had a negative impact on dentin elastic behavior [15, 21]. These studies immersed dentin bars in heated NaOCl, guaranteeing a stable temperature over an extended period of time. In the present study, however, heated NaOCl was delivered into the canal to simulate the clinical situation. In such a scenario, the temperature of consecutive amounts of heated NaOCl solution loaded into canal drops cannot be sustained at its extra-oral heated temperature of 70 °C [12].

Certain evidence has suggested that bacteria in mature biofilms are more resistant to 1% NaOCl than to 5.25% NaOCl. Effective removal has required adjunct activation techniques, such as ultrasonics [24] or Er,Cr:YSGG laser-activated irrigation [25]. While one study found that heating NaOCl to 37 °C had negligible effects on bacterial cell viability in a biofilm [26], another study found that 1% NaOCl at 45 °C dissolved pulp tissues as effectively as the 5.25% solution at 20 °C [10], in addition to demonstrating significant biofilm antibacterial efficacy.

Research has confirmed that NaOCl heated extra-orally to 70 °C will remain at ∼45 °C during canal irrigation for a few seconds [12]. With intracanal heating techniques such as heat pluggers, it is possible to deliver more consistent heating sustained between 40 and 49 °C [12]. This supports the use of NaOCl at a lower concentration without compromising its ability to dissolve organic tissue.

One disadvantage of using heated NaOCl for root canal irrigation is its association with higher postoperative pain and an increase in patient analgesic intake [27]. One possible explanation for this is heat promotion of the vasodilation inflammatory response. Another disadvantage is its possible adverse effect on the cyclic fatigue resistance of nickel titanium files used in root canal preparation [28, 29].

Although the present study mimicked the clinical situation in terms of dentin surface exposure, exposure time, and temperature, there was limited standardization of the dentin disks. The dentin disks had different diameters and canal sizes, which hindered the exact calculation of their compressive strengths. The orientation of the dentinal tubules was not taken into consideration, which may have affected the results. Specimens whose tubules’ planes are parallel to the exerted loads tend to yield results indicating more strength [30]. Although compressive strength tests used in the present study are less affected by specimen preparation flaws [31], the age, type, and storage time of teeth were not considered. These factors would have affected the inherent strength of the dentin, irrespective of endodontic treatment [32]. The pre-existing flaws in normal dentin affect its fracture resistance. These flaws propagate under consecutive loading cycles, leading to failure. The sectioning technique used in this study should also be considered. Although care was taken to eliminate disks with visible cracks, the presence of invisible flaws could not be excluded. Finally, understanding how the chemical environment to which the dentin is subjected during root canal preparation affects the growth of cracks is essential for formulating long-term scenarios [31]. Perhaps further studies taking these factors into account could help to verify the present findings in addition to employing non-invasive methods, such as finite element analysis.

## Conclusion

Within the conditions of this study, irrigation with NaOCl at different concentrations and temperatures during root canal preparation did not affect the compressive strength of root dentin.

### Electronic supplementary material

Below is the link to the electronic supplementary material.

Supplementary Material 1. S1 file contains the whole dataset.

## Data Availability

The datasets generated and analyzed in this study are available as a supplementary file.
